# Rice Farmers’ Perceptions and Experiences of Exposure When Storing, Using, and Disposing of Insecticide and Implications for Policy Development for Its Safe Use

**DOI:** 10.1177/10482911261448616

**Published:** 2026-05-08

**Authors:** Nishikanta Kumar, Valerie Nie, Kavitha Palaniappan, Joanna Bohatko Naismith

**Affiliations:** 1 5982 School of Health Sciences, University of Newcastle, Newcastle, Australia

**Keywords:** environmental harm, human health risks, insecticide use, rice farmers

## Abstract

Improper and unsafe insecticide handling in India remains a concern due to limited awareness and training, despite existing regulations. Using a phenomenological approach, the study gathered insights through semistructured interviews with seven farmers and farm workers in Odisha, India. The study identified environmental change, increasing crop vulnerability to pests, financial burdens, and modern cultivation methods as drivers leading to increased usage of insecticides. Farmers often relied on vendors for guidance rather than label instructions, highlighting the need for clearer communication and improved presentation of instructions to aid comprehension. Disposal practices such as releasing contaminated water into fields or canals and discarding containers by burying, burning, or selling pose risks to human health and the environment. The findings indicate there are systemic barriers to good practice, such as inadequate training or education, a lack of enforcement of regulations on safe handling, and a lack of infrastructure and financial resources for pesticide safe use and disposal. Addressing these issues requires comprehensive reforms, such as stronger policy implementation, more effective enforcement mechanisms, and sustained awareness-raising initiatives. Specific policy recommendations include clearer insecticide labeling, mandatory PPE provision, vendor training, promotion of Integrated Pest Management (IPM) and biopesticides, safe disposal practices, and stronger enforcement of pesticide regulations.

## Introduction

Agriculture is a crucial sector of the Indian economy, and productivity is enhanced by the use of a range of chemicals, in particular insecticides.^1^ In rural India, agriculture serves as the primary source of income for approximately 70% of the population.^2^ In 2020, 61,702 tons of pesticides were used in agricultural crop production in India.^3^ However, managing the safe handling of insecticides in rural communities poses significant challenges, raising serious concerns about associated environmental and human health impacts.^4^ Insecticide exposure can lead to acute illness, including symptoms such as headache, skin and eye irritation, nausea, vomiting, tremors, dizziness, and muscle weakness, and in severe cases, coma. Long-term exposure has been associated with chronic conditions such as cancer, neurological disorders, reproductive, and developmental issues.^5,6^ Environmentally, insecticides can harm many beneficial bacteria in soil, destroy the biodiversity of an ecosystem, and contaminate aquatic life.^7,8^ Systemic barriers, including limited knowledge and awareness, inadequate training and education, financial constraints, restricted access to essential resources and disposal infrastructure, and entrenched cultural and behavioral practices, can significantly impede farmers’ capacity to apply insecticides safely.^5,9–11^

Rice crops are prone to pest attack.^12^ This is partly related to the usage of High-Yield Variety (HYV) seeds, which boost crop yield but may lack resistance to attack by common pests, especially insects. In addition, changing temperatures and rainfall patterns are thought to favor the growth of pest populations, resulting in more reliance on using insecticides to maintain productivity.^13,14^

Improper insecticide usage remains widespread among rice farmers in India.^6^ Its persistence may reflect broader systemic challenges rather than isolated knowledge gaps. Incorrect application and mixing, unsafe disposal of containers, and insufficient PPE use may indicate weaknesses in policy implementation, enforcement mechanisms, and training on safe handling practices.^5^ Furthermore, a reliance on informal sources of information, such as the local pesticide vendor or representative, rather than technically correct, documented instructions for the safe application of insecticide, may result in misinformation being promulgated. This may increase the risk of insecticide exposure.^15–17^

The Indian Government has established several legislative instruments to regulate the safe use of pesticides in agriculture, most notably the Insecticides Act of 1968 and the Insecticides Rules of 1971. These laws govern the manufacture, import, sale, transport, distribution, and use of insecticides. In addition, they mandate the packaging and registration of insecticides, as well as labeling with safety instructions.^18,19^ Other relevant legislation includes The Environment (Protection) Act,^20^ The Hazardous and Other Wastes (Management and Transboundary Movement) Rules,^21^ The Occupational Safety, Health and Working Conditions Code,^22^ and The Pesticide Management Bill.^23^ This bill is broader in focus than the Insecticides Act and Rules, and it includes consideration of compliance and enforcement.

In India, agricultural safety enforcement is fragmented across multiple ministries. There is no Central Government body equivalent to SafeWork Australia^24^ or the Health and Safety Executive in the United Kingdom,^25^ which has primary roles in workplace health and safety. These bodies disseminate legal requirements and provide information and guidance on how to meet them. It is unclear how effectively the Indian legislation protecting worker health, including agricultural worker health, is implemented.^26^ Furthermore, there is still a lack of clarity regarding the effectiveness of procedures for monitoring pesticide use and enforcing compliance with the legislation.^27^

Several quantitative studies have examined insecticide usage practices and associated health symptoms among farm-working communities in India.^5,6,12^ However, these studies did not capture the complexity of human perceptions, behaviors, and attitudes, nor any systemic reasons for them. Qualitative research helps bridge this gap by offering deeper insights into personal experiences and perception, thereby enriching our understanding of the complex social and cultural dimensions surrounding insecticide use, leading to a deeper understanding of the issues. A phenomenological methodology was employed in this study to explore, describe, and interpret human behavior through the lens of individual lived experience.

The present study aims to investigate the perceptions, experiences, and practices related to insecticide use, encompassing storage, mixing, application, and disposal, among rice farmers and farmworkers in Odisha, India. It seeks to identify the social and environmental factors that influence adherence to protocols and instructions for the safe usage of insecticides, which need to be understood to develop effective policies and procedures for the safe use of insecticides in agriculture.

## Method

A phenomenological approach was used in this study to gain an understanding of the experiences and perceptions of insecticide usage and associated health effects of rice farmers and farmworkers from Odisha, India.^28^ The study adopted the Standards for Reporting Qualitative Research (SRQR), developed by O’Brien, to ensure transparency in reporting.^29^

SRQR provides a structured set of guidelines for reporting qualitative studies, including clear descriptions of the research design, participant selection, data collection procedures, data analysis, and strategies used to ensure the credibility and rigor of the findings. These standards also promote consistency in qualitative research reporting and enable readers to better evaluate the methodological rigor, credibility, and transferability of the study findings. The study employed a semistructured interview design, characterized by a predetermined set of open-ended questions. This approach was selected to facilitate discussion and ensure consistency, while also allowing the interviewer to delve into specific themes or responses as they emerged.^30^ Questions derived from key literature^5^ were used to elicit information about the participants’ experiences and perceptions of insecticide use, exposure, and health effects. Each participant was given the option to terminate the interview at any point; however, all participants completed the interviews. Seven rice farmers and farm workers were interviewed for this study. The number of interviews was determined by the recognition of information redundancy, which occurs when no new data emerge from the interviews.^28^ According to Morse,^31^ when the topic is clear and the required information is easily obtained, fewer participants are needed. Ethical approval for the study was granted by the University of Newcastle, Human Research Ethics Committee (Ethics Number: H-2022-0239).

### Participants

Purposive sampling was used to recruit rice farmers and farmworkers. Potential participants were selected from a larger cohort that was participating in a field study being conducted in Odisha, India. This sampling method identified participants likely to provide rich, in-depth information on the topic under investigation.^32^ While it is arguable that this study sample does not represent all rice growers, it does offer perspectives on the experiences of a range of farmers and farm workers. The inclusion criteria for a participant in the study were as follows:

1. Must be a farmer or farmworker, male or female, aged 18 years and over,
2. Engaged in rice cultivation, using insecticides on the rice crops on a minimum of one farm, and
3. Fluent in the native language, Odia.

### Recruitment

Farmers and farmworkers attend the local village Panchayat Office in Odisha, India, each month, to collect their personal rice rations. Approval to recruit potential study participants at the Panchayat Office was obtained from the Chief District Agricultural Officer, Bargarh, Odisha. Each village is notified in advance of the exact date for distribution of the rice rations at the Panchayat Office, enabling the researcher to attend on the same date. Following the completion of the survey, an information statement detailing the study and a consent form were provided to interested participants. Each consenting participant then contacted the researcher to schedule a convenient time and location for the interview.

### Data Collection

Interviews with participants typically lasted 30 min, allowing sufficient time for the topic to be explored. Each interview was conducted at the participant's farm or home, between October and December 2022. Basic demographic data on occupation, sex, age, literacy, and education were collected for each consenting participant. Before the interview began, potential participants were invited to ask the researcher questions and to raise any concerns they might have about the interview. Participants were informed of their right to withdraw from the study at any time and/or to withdraw their data. Confidentiality and anonymity were discussed, and participants were informed that the interview would be recorded for accurate transcription. Pseudonyms were used to deidentify participants in the transcriptions. During the interviews, farmers and farm workers were asked to describe their experiences with insecticide usage, exposure, and any health effects they may have experienced.

### Data Analysis

Interviews were audio recorded and subsequently transcribed verbatim by the researcher (NK). The final transcripts were translated into English for analysis. Participants were offered the option of member checking, also known as participant or respondent validation, a technique used to explore the credibility of results.^33^ However, no participant accepted the invitation.

An inductive approach, reflexive iteration, was used to analyze the data. Reflexive iteration, as described by Srivastava and Hopwood,^34^ is an important method for evoking insight and developing meaning. This iterative process involves researchers repeatedly reviewing the data to connect it with emerging insights, progressively leading to a more refined focus and understanding.^34^

Initially, two researchers reviewed the transcripts and identified codes and categories that best described the experiences of the participants. The codes were initially categorized into themes, which were subsequently refined into three key themes. Trustworthiness was established through peer debriefing, whereby the research process, data collection procedures, and preliminary interpretations were discussed with academic peers and supervisors to obtain critical feedback, ensure methodological consistency, and minimize potential researcher bias. This manual process allowed for a deeper engagement with participants’ narratives and ensured that the analysis remained closely connected to the original data. All data collected from this study are stored on the University of Newcastle, Australia's secure IT database, OwnCloud.

## Results

### Demographics

Semistructured interviews were conducted with seven male rice farmers and/or farmworkers from Odisha, India. Table 1 describes the demographic characteristics of individual participants. All participants were over 18 years of age, and some were over 60. All were fluent in Odia, their native tongue. Participants completed their schooling between the 9th and 12th standards. Typically, the 9th standard corresponds to age 14 years and the 12th standard to age 18 years. One participant had completed a master's degree.

**Table 1.** Demographic characteristics of study participants.

Participants	Types of participants	Age range	Ability to read and write	Education completed
Participant 1	Farmer	60 and above	√	Master's degree
Participant 2	Farmer	30–39	√	10th
Participant 3	Farmer	50–59	√	10th
Participant 4	Farmer^a^ and farm worker^b^	60 and above	√	12th
Participant 5	Farmer and farm worker	30–39	√	12th
Participant 6	Farmer and farm workers	18–29	√	9th
Participant 7	Farmer and farm workers	50–59	√	12th

a A farmer (also called cultivator or operational holder) is a person/household who has operational control over agricultural land and exercises management decisions regarding cultivation.

b A farm worker (agricultural laborer) is a person primarily engaged in agricultural wage work on others’ farms and who does not operate land or operates only a negligible area.

### Key Themes Emerging From Transcript Analysis

Analysis of the transcripts identified three key themes on rice farming in India. These themes were:

1. Increasing insecticide usage,
2. Mixing and applying granular or liquid insecticides and
3. Cleaning and disposing of insecticides.

### Increasing Insecticide Usage

(a) The interview responses indicated that the farmers and farmworkers who participated in this study attributed the increasing use of insecticides in agriculture to several factors:
  - Environmental changes,
  - the necessity for insect and disease management,
  - financial pressures,
  - the adoption of modern agricultural practices, and
  - the challenges faced in providing and promoting safe usage instructions for insecticides.

Most respondents believed that the impact of environmental change, characterized by frequent and unpredictable variations in weather patterns, and the extensive use of various pesticides, especially insecticides, contributed to the development of increased pesticide-resistant insect populations in rice paddy fields. They noted that addressing this issue often necessitated a gradual escalation to applying larger insecticide doses.

Agricultural officers mostly blame the weather for an increasing rate of insects in the crop field. (Participant 5)

Four participants reported that increased insect infestation resulted in increased insecticide use, as described by Participant 2 below.

The over-application of insecticides escalates in the increasing prevalence of different kinds of insects and diseases in rice farms.

(b) Financial burden and modern agricultural production

Four participants observed that farmers bear a huge responsibility for the welfare of their family members, as per local cultural customs. Their primary income is typically derived from agricultural production. The financial burden on the farmers necessitates a more profit-oriented approach to paddy crop production, which includes the adoption of modern cultivation practices. These practices involve increased pesticide application rates, the use of HYV seeds, and the implementation of modern farming machinery.

We were buying kerosene at 2 to 3 rupees per liter as our energy and lighting source, but it is no more available. Now we pay the electric bill for an average of 500 Indian rupees per month. Before, we used wood for cooking food, but these days, gas became compulsorily used, which costs around 1000 rupees per month. Therefore, we have to use different types of insecticides for increase production to manage our daily expenses. (Participant 6)

Participants 2, 3, and 7 made similar statements, and Participant 3 added:

With the introduction of modern ways of cultivation with the usage of high-yield seeds and pesticides, the production level of rice crops has enormously increased.

(c) Challenges in providing and promoting safe usage instructions

Most of the farmers mentioned that they encounter many challenges in the safe use of insecticides. A significant issue highlighted by participants is the overuse of insecticides, driven by the belief that higher doses will more effectively protect crops from pests and thereby increase production. Additionally, the persistence of traditional cultivation practices and peer influences are consistently perceived as factors contributing to the neglect of safe usage practices, such as wearing personal protective clothes and washing hands after insecticide application.

Participant 3 highlighted in his interview that:

If you don’t apply higher doses, you can’t control highly resistant insects. As a result, people are applying very powerful medicines and increasing the quantity of their applications.

Participant 1 further added:

They think the insecticides will not harm their health, and the influence of surrounding farm workers not using protective clothes is also a crucial reason for not following safe protection methods.

### Mixing and Applying Granular or Liquid Insecticides

All farmers and farmworkers reported employing different methods for mixing and applying granular and liquid insecticides.

(a) Granular insecticide mixing and application practices and tools

All participants explained that granular insecticides are mixed with fertilizers, sand, or other solid substances and then spread on large polythene or plastic sheets laid on the ground, before application. Such traditional practices ensure proper mixing and uniform application on the farmland. These practices reflect farmers’ commonly followed preparation methods as described during the interviews, although the study did not assess whether they fully comply with manufacturers’ instructions. The participants also reported that gloves are rarely used during this process. They also indicated that manufacturers, distributors, or employers generally do not supply the type of gloves specified on pesticide labels, and farmers typically obtain protective equipment on their own, often resulting in the use of inadequate or no hand protection. As Participant 3 reported:

The granular insecticides are applied by hand after properly mixing with sand or fertilizers. They apply it without wearing gloves, but wash their hands with soap after the application procedure.

(b) Liquid insecticide mixing and application practices and tools

All participants in the study reported that they typically prepare insecticide solutions in buckets by mixing liquid insecticides with water sourced from the paddy fields. These buckets are commonly made of iron, steel, or plastic (often repurposed fertilizer storage containers), and all of them are frequently reused for other household purposes, such as storing water for bathing. As Participant 4 reported:

After completion of insecticide mixing, we usually wash that bucket and bring it back home for use again for general purposes.

However, some participants reported using machines for spraying insecticides, either motorized sprayer machines or manually operated hand-pumping machines. Many participants reported mixing liquid insecticides with water directly in the spraying machine, without the use of a bucket.

Participant 4 stated:

As per the quantity specified on the pesticide labels, we mix accordingly 15 ml, 20 ml, or 40 ml with 15 liters of water in the spraying machine.

Other mixing methods reported include using measuring cups to combine insecticides with substances such as gums or shampoos, to enhance their effectiveness. This practice enhances the adhesive properties of the insecticide mixes, enabling them to adhere to rice crop leaves, rather than fall to the ground. Participants reported that in some instances, insecticide sprayers apply the insecticides directly to the crops, without dilution with water. These practices are not typically specified in manufacturers’ instructions. Participant 2 articulated this practice as follows:

Liquid insecticides require water to be mixed before application, although in certain cases, the insecticide application process is conducted without mixing it with water.

Participant 5 stated:

The liquid insecticides are mixed with the farm water in a bucket, and then shampoo or gums are added before application.

(c) Adherence to instructions and vendor guidance

Many farmers reported that they and other insecticide users generally adhered to the written instructions on insecticide containers. However, some indicated that they either ignored the instructions or were unable to follow them, due to a lack of written guidelines on the labels, or to guidelines being written in a language not comprehensible to the farmers. Although the instructions were often provided in multiple languages, most farmers and farmworkers relied on vendors for advice on usage practices. Participants noted that farmers with lower literacy or education levels sought guidance from vendors or local representatives on the correct application methods. Participant 2 expressed this as:

We can apply insecticide by reading the instructions on the bottle as well as the instructions given by the vendor to apply doses in the agricultural field.

c

Clean up and disposal of insecticides: All participants suggested that a high priority was cleaning insecticide spraying machines after completion of the application. The farmers and farmworkers explained that they and other insecticide users adopted various methods to clean their insecticide containers and spraying machines. Most of them used water alone from nearby irrigation canals, or water with detergents and sand, to clean the spraying machines. After cleaning, the detergent-laden water was often discharged near the crop fields or into nearby canals. Following the cleaning process, the spraying machines were typically dried using sunlight. Participant 7 expressed his view that

After spraying, we wash the spraying machines with water and detergent. The detergent water is drained into canals, fields, or farm water.

Participants highlighted the importance of thoroughly cleaning, rinsing, and draining insecticide bottles before disposal. However, this meticulous process often did not extend to the adoption of safe disposal methods. Disposal practices varied significantly among farmers, with many reporting that they discarded bottles in the crop fields or left them exposed on open ground. Alternative disposal methods included burying, burning, or selling the bottles to local waste collectors. The prevalence of these inappropriate disposal practices was primarily attributed to a lack of awareness or inadequate instructions regarding correct disposal procedures. As Participant 2 explains:

In most cases, the bottles are disposed of through burning or burying, or by keeping in the storage place.

Further, Participant 4 said that:

Most people have no idea about the disposing method. After application, they throw the empty bottles in the side areas of the agricultural field. If it is an aluminum bottle, then farmers bring it back home to sell.

In addition, farmers and farm workers reported repurposing cleaned empty insecticide containers for other uses, such as storing food items, using them as measuring cups for mixing insecticides prior to application, or repurposing them as pots for planting. Participant 2 explains this process:

The empty bottles are disposed of or brought back home to keep them safe, and in some cases, it is used for selling. In some other cases, after washing an empty bottle, it is used as a measurement cup for mixing insecticides.

Participant 1 also elaborated:

Some people use the empty insecticide bottle to store cumin powder, coriander powder, or chili powder. Some people bring it home and sell it to waste pickers to exchange the bottle for eggs.

## Discussion

This study is the first to explore the perceptions and experiences of insecticide use in rice farmers and farm workers in Odisha, India. It offers valuable insights into the increasing usage of insecticides and the practices of mixing, application, cleaning, and disposal employed by these rice farmers and farmworkers.

Rice farmers and farmworkers in this study reported that environmental changes, the development of pesticide-resistant insect populations, financial pressures on farmers, and modern cultivation methods were key factors directly or indirectly contributing to the increased use of insecticides.

A recent study in 2023 reported that rising temperatures and changing precipitation patterns significantly influence the dynamics of increasing pest populations on farmland.^14^ Higher temperatures can influence insect growth rate, metabolism, development, and reproduction, resulting in prolonged life cycles and the likelihood of multiple generations within a crop cultivation period.^14^ Heavy precipitation can decrease pest populations, whereas drought conditions can increase them, favoring pest attacks on crops. The rapid increase in pest populations on rice farmland significantly contributes to the excessive use of insecticides.^14^

The current study highlights that financial burdens can also contribute to increased usage of insecticides. A literature review conducted by Wilson and Tisdell in 2001 emphasized the “economic pressures” faced by farmers, particularly the requirement for expenditure to support the family on a limited income. The review determined that economic pressure is a fundamental driving force behind farmers’ reliance on pesticides and insecticides to maximize productivity.^35^ Similarly, a study from Brazil identified the primary factor influencing pesticide usage to improve crop yield was the family's financial situation.^36^

Another significant factor highlighted by the farmers in this study is the influence of modern cultivation practices on increased usage of insecticides. The use of HYV seeds necessitates greater insecticide application, due to their higher susceptibility to pest attack. Although HYV seeds are developed primarily for high-yield production, they often lack inherent pest resistance, thereby requiring extensive pesticide use to manage insects and other pests and enhance yield.^13^

The current research identified practices of combining various complex substances with granular insecticides before application. Participants described the process of mixing granular insecticide with fertilizer, sand, or other substances prior to application on rice crops. When insecticides are combined with other substances such as fertilizers, new chemical interactions may occur, potentially reducing the effectiveness of the insecticides.^37^ The extent to which these potential interactions reduce the effectiveness of granular insecticides in practice is not known, nor is the effect of mixing liquid insecticide with gums and shampoos, as reported by many participants. These practices represent farmers’ reported behaviors rather than recommended or label-specified application methods.

In the case of liquid insecticide preparation, participants in this study used buckets to mix the insecticide with water. Buckets were made of iron, steel, or plastic and were frequently repurposed for household tasks, such as storing water for bathing. Although some suppliers or fellow farmers verbally advised farmers and farmworkers not to reuse pesticide containers or buckets, guidance was inconsistent and informal. Suppliers are not legally required to provide such information, and without clear instructions or alternatives, many farmers continued to repurpose buckets for household use.

Previous research has reported other farmers using buckets for insecticide mixing and daily activities.^38,39^ Repurposing buckets previously used for mixing insecticides to store water may increase the risk of insecticide exposure through the skin, especially during bathing.^38,39^ This practice emphasizes the urgent need for awareness programs and the promotion of proper disposal practices, such as triple-rinsing, puncturing containers to prevent reuse, and returning them to designated collection or take-back facilities, to mitigate health and environmental risks.

While awareness programs are important for improving farmers’ knowledge of safe practices, they are not sufficient on their own, and systemic factors behind unsafe pesticide use, which include economic constraints, lack of protective equipment, limited training, and unsafe storage practices, need to be addressed.^38^ Policy recommendations to address these issues include providing affordable protective gear, training programs, regulated pesticide use, safe storage facilities, and strengthened community health services. There is also a need for clearer accountability within the supply chain, including legal requirements for suppliers to provide accurate safety instructions and guidance. Holding suppliers responsible for providing this information, as well as making them liable for all downstream damage, would help ensure farmers receive consistent and reliable advice at the point of sale.

Farmers in this study reported frequently seeking advice on insecticide use from pesticide vendors and local representatives, relying on their knowledge and experience instead of the written instructions on insecticide containers, which were often poorly printed or entirely absent. Studies conducted in Africa have shown that a lack of written guidelines on insecticide use, or the presentation of guidelines in foreign languages, creates challenges for farmers attempting to read, understand, and adhere to safe insecticide application processes.^16,40^ A study from Nepal in 2021 further highlighted farmers’ heavy reliance on pesticide vendors for guidance on insecticide application, particularly when multiple-language instructions were absent from insecticide labeling.^17^ This reliance may indicate that farmers place more trust in vendor advice than in written instructions, reflecting a lack of confidence in formal written guidelines. A continual reliance on vendors could limit understanding of the potential health and environmental hazards associated with improper pesticide application, which may be exacerbated by illiteracy and inadequate training on safety practices.^9,41^

These issues highlight the need to establish clearer legal responsibilities for vendors, including requirements to provide accurate safety information at the point of sale, explain proper handling and disposal procedures, and ensure that farmers receive basic guidance regardless of literacy level. Such obligations would help reduce over-reliance on informal advice and promote safer pesticide use.

The practice of using detergent or water for thorough cleaning and rinsing of the spraying machines after completing pesticide application has been reported as being quite common.^42^ However, the detergent-laden water often drains into the crop fields, canals, ponds, rivers, and open ground, which can lead to environmental contamination, particularly affecting the soil and water quality.^43^ In this study, after cleaning spraying equipment and machinery, farmers typically discharged the washing water, presumed to be contaminated with insecticide, into nearby crop fields or canals. Farmers reported disposing of empty insecticide containers by discarding them in the crop fields, burying them in soil, burning them, or selling them to local waste collectors. Discarding potentially contaminated insecticide containers in the fields presents a public and environmental health risk. In addition, one study has reported that burning pesticides can release toxic chemicals into the atmosphere, leading to air pollution and potential health risks to humans.^44^ Over time, airborne toxins gradually settle on soil and water, resulting in prolonged environmental contamination.^45^ Similarly, burying insecticide bottles in soil can lead to toxins leaching into the soil and groundwater, thereby polluting drinking water and farmland. This contamination can disrupt ecosystems, destroying microorganisms and impacting fauna through the food chain.^46^

At present, farmers have no access to designated facilities for safely washing and spraying machinery. Establishing a safe method would require investment in infrastructure, such as controlled wash stations and wastewater treatment systems, together with clear guidelines for their use and supported by a regulatory framework. Given the economic importance of insecticide use in rice growing, the development of such infrastructure is essential to reduce environmental contamination and support safer farming practices.

Unsafe disposal practices also highlight the absence of a structured system for managing used pesticide containers. Beyond farmer-level behavior change, there is a need for a shared responsibility model in which vendors are required to take back used containers, and manufacturers are obliged to retrieve them from vendors for safe recycling or disposal. Such extended producer responsibility (EPR) initiatives would reduce the burden on farmers who currently have no safe disposal options and would prevent further contamination of soil and groundwater.

The findings of this study suggest that farmers’ perceptions of health and environmental risks and their behaviors when handling insecticides in part contribute to the continued unsafe practices being widely adopted in the community. Understanding this relationship between the perceptual framework and practice is important in developing policies and programs for the effective provision of information on, and training in, safe insecticide use in rice farming, and in the development of legislation to underpin it. While farmers’ perceptions and behaviors clearly contribute to continued unsafe practices, it is important to recognize that many of these behaviors occur in the absence of any safe disposal infrastructure. Even when farmers are aware that available methods of container cleaning and disposal are unsafe, they often have no alternative options. Therefore, improvements in knowledge and training must be accompanied by broader structural and policy interventions, including investment in appropriate waste-management infrastructure and clear regulatory mechanisms to enable farmers to adopt safe practices in a practical and sustainable way.

In India, the Insecticide Act, 1968,^18^ and the Insecticide Rules, 1971^19^ regulate the manufacture, sale, transport, and safe usage of insecticides to mitigate human, animal, and environmental health risks. They also describe registration and licensing of manufacturers and sellers, and safety guidelines for packaging and labeling pesticides. Despite the longstanding establishment of this Act and its accompanying Rules, enforcement at the village level remains significantly inadequate, particularly in rural areas where the majority of rice cultivators reside. Moreover, despite the legislative requirements, pesticide labeling is sometimes absent or unclear, possibly contributing to reports by farmers that they relied upon local pesticide vendors for information about safe application procedures, rather than on instructions printed on the bottle. This reliance may have contributed to the unsafe insecticide practices observed among the farming population in the current study.

The Insecticides Act (1968) and the Insecticides Rules (1971) place explicit obligations on manufacturers and packers to provide labeling, instructions, and warnings on insecticide containers and require directions for “safe disposal of used containers.” The Rules require inscriptions such as “Dangerous to re-use empty containers.” Other key provisions include mandatory safety leaflets with application instructions and poisoning antidotes (Rule 18), safe disposal of surplus pesticides, containers (to be broken and buried), and how equipment is to be washed to prevent pollution (Rule 44). Detailed labeling requirements include toxicity symbols, color codes, warnings, and multilingual text (Rules 16–19). Mandatory PPE can include impermeable clothing, gloves, goggles, and respirators during handling (Rules 39–40). In addition, the Act and Rules empower authorities to require measures for worker safety and training. These provisions therefore create a clear regulatory basis for (a) vendor/manufacturer labeling and instructions and (b) employer duties in the production/handling context. However, these provisions are inadequate in several respects: they lack mandatory on-farm training or dosage verification, fail to mandate climate-appropriate PPE for farmers, and provide only general disposal guidelines without monitoring mechanisms. They permit small fonts or complex labeling that reduces accessibility for low-literacy users.

The Pesticide Management Bill (PMB) was developed to replace the earlier legislation and is more comprehensive in its coverage.^23^ It considers both occupational and environmental health and addresses monitoring of compliance and enforcement. The bill promotes the usage of biopesticides and Integrated Pest Management (IPM). It requires pesticide retailers to be trained professionals, ensuring safe dispensing practices, and includes measures to regulate sales and distribution. It contains provisions for registration and traceability that aim to reduce unsafe or unlabeled products entering the market, and specific provisions that require or enable capacity building, such as training and certification for retailers. Many of these features directly target problems we observed (poor labeling, untrained vendors, weak enforcement) though, as noted, the Bill's practical effect at the village level depends on operational rules, notification dates, and enforcement resources. However, the Pesticide Management Bill omits specific mandates on PPE, spray device washing, detailed labeling standards, and container disposal protocols, relying instead on advisory roles for the Central Pesticides Board.

The PMB was only enacted in 2020, so its impact on pesticide safety amongst end-users has not been fully investigated. Furthermore, the PMB had only just come into force at the time of data collection in the current study.

In practice, implementation of these provisions remains highly inadequate across India. Weak enforcement due to limited inspectors, infrequent field monitoring, and resource constraints has resulted in widespread misuse, overdosing, improper container disposal (often reused or discarded openly), contamination from equipment washings, and negligible PPE adoption among farmers. Labeling information is frequently ignored as farmers depend on dealer advice rather than reading complex or incomplete labels, while training programs (e.g., in integrated pest management) have reached only a small fraction of the farming population. These gaps contribute to persistent occupational poisoning, environmental contamination, and pesticide residues in food, underscoring that both the existing Act/Rules and the pending Bill require stronger mandatory safeguards, rigorous enforcement mechanisms, and farmer-centric education to effectively mitigate pesticide-related risks.

The evidence presented here suggests that gaps remain in the dissemination of legislation, in the provision of guidance on implementation and compliance, and in the adoption of safe practices in using pesticides. Specifically, the principal gaps are: (a) labeling—occasional absence or illegibility of instructions on product containers despite legal requirements (Rules 1971); (b) retailer training and behavior—widespread reliance on vendors for spray advice in lieu of written instructions; (c) disposal practice—reuse or improper dumping of empty containers despite mandatory printed disposal warnings; (d) local enforcement and outreach—limited active monitoring by agricultural officers and scant farmer training programs; and (e) infrastructure—lack of local collection/processing facilities for contaminated containers.

### Recommendations for Policy to Improve the Health and Safety of Pesticide End-Users

The primary recommendations for policy improvements are:

- Clear and understandable labeling on insecticide bottles:

Many farmers reported difficulty understanding instructions due to poorly printed or absent labels, leading them to rely on vendors for advice. A comprehensive policy should be developed to mandate that all insecticide containers feature clear multilingual labels, with priority given to local languages to ensure accessibility. For individuals with limited literacy, these labels should be supplemented with intuitive pictorial guides to effectively communicate safe handling practices and reduce the risk of misuse. Pictorial guides are an evidence-based, low-cost tool that increases comprehension among low-literacy farmers, but are most effective when used alongside short, practical demonstrations and supervised practice delivered by trained extension workers.

Multiple agricultural-education studies show pictorial/illustrated materials substantially improve comprehension among low-literacy farmers and increase correct behavior (label reading, PPE use) when combined with brief interpersonal training.^47^ Pictorial guides are low-cost and effective as a complement to verbal training and demonstrations. While not a silver bullet, pictorial labeling plus outreach demonstrably improves safer handling and storage. Short, practical trainings (half day to 2 days) are necessary to translate pictorial cues into correct practice. Key components include how to read labels and pictograms, correct mixing and spray technique, PPE donning/doffing and maintenance, triple-rinse and container disposal, first-aid and emergency contacts, and IPM basics. Practical demonstration and supervised practice are essential.^48^

- Regulatory reforms for mandatory PPE provision

It is not currently possible to develop engineering controls for pesticide exposure in these rice-growing farms in India, partly because the location is not fixed and consists of open fields, but also because of financial constraints in largely subsistence farming. Accordingly, there is a heavy reliance on PPE control. However, manufacturers could potentially be required to supply pesticides through closed transfer systems as a condition of market access in India, which may help reduce exposure risks at the source. However, implementation would depend on regulatory enforcement, affordability, supply chain capacity, and the suitability of such systems for small-scale farming contexts. Many farmers reported routinely mixing pesticides with their bare hands and applying them without gloves. This dangerous practice stems largely from the fact that PPE is not provided with pesticides, and cost-burdened farmers cannot afford to purchase it separately. The Insecticides Act of 1968 and the proposed Pesticide Management Bill 2020 fail to explicitly require manufacturers, distributors, or employers to provide label-specified PPE to pesticide applicators. Therefore, policy recommendations should include:

1. legally requiring pesticide manufacturers and distributors to supply climate-suitable PPE free of cost with every pesticide purchase, matching label specifications for glove types and protective gear;
2. mandating farm owners and contractors to provide, maintain, and replace PPE for all workers;
3. establishing compulsory training and certification programs for all pesticide applicators on proper PPE use;
4. implementing strict enforcement mechanisms with substantial penalties for noncompliance;
5. creating a compensation fund for affected workers, financed through levies on pesticide sales.

- Provide training to certified vendors:

Farmers heavily relied on pesticide vendors for instructions, often due to a lack of confidence in or access to written guidelines. Due to the heavy reliance of farmers and farmworkers on vendors for guidance, it is essential to mandate certified training for all pesticide vendors on the safe use of insecticides. Vendors should be required to provide accurate, context-appropriate information, advice, and materials in the local language, to ensure that farmers and farmworkers can make informed and safe decisions regarding insecticide use. This could be achieved through the enforcement of PMB provisions to regulate retailers and by a legal requirement for retailer certification, plus mandatory provision of label-based guidance and safety demonstrations at the point of sale.

- Promote integrated pest management (IPM) and biopesticide use:

Farmers reported increased insecticide use due to pest resistance and the vulnerability of HYV seeds to insect attacks. Farmers and farmworkers should be incentivized to adopt Integrated Pest Management (IPM) and biopesticide practices. This transition would not only reduce environmental harm but also promote more sustainable agricultural methods.

- Safe insecticide disposal practices:

Farmers reported disposing of washing water and empty insecticide containers in crop fields, canals, or by burning, posing serious environmental risks. To mitigate environmental contamination, it is essential to establish facilities for insecticide container disposal, along with designated areas for draining and washing residual insecticides near agricultural fields and waterbodies. Draining and washing facilities are integrated into the designated collection sites, with appropriate containment measures (e.g., concrete flooring and bunding) to capture residues. Pesticide-containing wastewater is collected and managed through controlled containment or evaporation systems, rather than being released into surrounding soil or water bodies. Designated collection sites should also be simple but secure, labeled drums for rinsed containers, PPE and spill-response kits, signage, and documented transfer arrangements to regional disposal facilities. However, implementing such infrastructure would require support from local or regional governments, as well as access to both technological and financial resources. The latter could include initial central/state funding for pilot programs, with consideration of an earmarked manufacturer/vendor levy, or an extended producer responsibility (EPR) funding scheme to sustainably fund collection, disposal, and training, all designed to protect smallholder affordability.

- Strict enforcement of legislation locally:

The study revealed inadequate enforcement of regulations governing insecticide use, leading to unsafe practices. Despite existing rules and the Pesticide Management Bill, aimed at ensuring the safe regulation of pesticide usage in India, enforcement remains weak at the local and village levels. To address this gap, local agricultural officers and panchayat bodies should be strengthened and adequately resourced to effectively monitor safe insecticide practices, including proper storage, disposal, and labeling. Specific relevant PMB provisions include strengthened registration and traceability (reducing circulation of unlabeled/illegal products), statutory emphasis on Integrated Pest Management IPM/biopesticides (minimizing hazardous product use), and measures to regulate and professionalize pesticide retailers, all designed to strengthen information flow and compliance from manufacturer to end-user. Strengthening safe insecticide use requires a combination of development of defined safe operating procedures, operational support (time and funds for outreach), monitoring tools, and community partnerships, so panchayats and agricultural officers can deliver training, inspect labels, and implement localized disposal solutions. Existing agricultural office staffing is insufficient for village-by-village outreach. We propose a certified modular training (2–5 days) delivered by an extension agricultural officer responsible for a cluster of villages and supported by trained local farmers designated as “safety champions.”

### Limitations of This Study

Although this study provides some insights into the experiences of farmers and farm workers in Odisha, India, it has certain limitations. The rice farming methods employed, the qualitative nature of the semistructured interviews, and the modest sample size all limit the generalizability and applicability of the findings to other types of farming and other countries. However, even though there were only seven participants, their interview responses did reach data saturation. In broad terms, all participants were reporting similar information. It is therefore probable that the findings could be replicated in a larger population of rice farmers in India. However, the policy recommendations presented in this study are specific to the local context of the study area and are not intended to be generalized to the national level. The qualitative methodology employed was appropriate for exploring the perceptions and experiences of farmers and farmworkers in India, who, under normal circumstances, rarely have the opportunity to express their opinions about their work. Additionally, although participants were offered the opportunity for member checking (respondent validation) to review and confirm the findings, none accepted the invitation. The absence of respondent validation may limit the ability to further confirm the interpretation of participants’ perspectives.

## Conclusion

This study provides a unique understanding of the perceptions, experiences, and challenges faced by rice farmers and farmworkers in Odisha, India, who are involved in the preparation, application, and disposal of insecticides. This study highlights key factors driving the increasing dependence on insecticides in rice farming. These factors include environmental changes, economic pressures, and modern agricultural practices.

Unsafe practices during mixing, application, cleaning, and disposing of insecticide containers were identified as serious concerns for human health and environmental contamination. Furthermore, the reported reliance on pesticide vendors for informal information on insecticide application procedures highlights the lack of adequate training. This, coupled with lower safety awareness among farmers and farmworkers’, highlights the necessity for targeted safety interventions, including investment in appropriate waste-management infrastructure and clear regulatory mechanisms to enable farmers to adopt safe practices in a practical and sustainable way. The findings of this study could have significant implications for regulators and government bodies involved in policy development for pesticide regulation, agricultural safety, environmental protection, and public health initiatives. Specifically, the findings highlight the need for stricter enforcement of pesticide handling guidelines, improved farmer education and training programs, and a reduction in environmental contamination resulting from unsafe insecticide use. Raising awareness of safe practices can assist with the promotion of sustainable agricultural methods and the mitigation of health risks to both humans and the environment. Improvements in policies governing pesticide use to provide a better framework for promoting their safe use, particularly in rice farming, were also discussed.
